# Supplementary material for: Using Andersen’s behavioral model of health care utilization in a decentralized program to examine the use of antenatal care in rural western Ethiopia
Source: PLoS One. 2020 Jan 27;15(1):e0228282. doi: 10.1371/journal.pone.0228282 (PMC6984696; doi:10.1371/journal.pone.0228282)
Supplement: S1 Text — (DOCX) [file pone.0228282.s002.docx]

**Household Survey Questionnaire for women of Reproductive Age**

**Addis Ababa University**

**College of Social Sciences**

Questionnaire designed to examine a study problem titled as “Using Andersen’s behavioral model of health care utilization in a decentralized program to examine the use of antenatal care in rural western Ethiopia”

**Consent form that certify the respondent’s agreement before the interview**

1. Name of the *Kebele*: __________________________________________
2. Questionnaire Identification Number: ________________________

**Introduction**

Good morning/afternoon. My name is _____________________. I am public health science professional and now I am collecting data from pregnant women of reproductive age groups (15- 49 years) for the research being conducted to identify factors affecting utilization recommended number of ANC visits and fewer than recommended number of ANC visits versus no visits as a base model, using Andersen’s Behavioral health care utilization in Gida Ayana *Woreda*, rural western Ethiopia Mr. Habtamu Tolera, and Professor Tegegne Gebre Egziabher from Addis Ababa University Social Sciences College and Dr. Helmut Kloos from California University, Department of Epidemiology and Biostatistics. You are selected to be one of the participants in the study by chance. The study will be conducted through interview. Your name and other personal identifiers will not be recorded on data collection format and the information that you give us will be kept confidential and will also be used for this study purpose alone. A code number will identify every participant and no names will be used. If a report of the result is published, only summarized information of the total group will appear. The interview takes 40 minutes on average and is voluntary and you have the right to participate, or not to participate or to refuse at any time during the interview. You will not face any problem if you do not agree to the information to be asked. Your participation on this study helps to improve and identify factors affecting recommended minimum number of ANC visits in the study *Woreda*. If you have any questions about this study you can ask me or contact the principal investigator Mr. Habtamu Tolera; Email Address: habtol@yahoo.com. Mobile: +251912015545. P.O.Bo:1176.

Are you willing to participate in the study?

1. Yes 2. No

Interviewer who certified that the informed consent has been given in written from the respondents

Name:__________________ Signature:__________________________Date:_______________

**Result**

1. Completed
2. Partially completed
3. Refused to complete

**Checked by:**

Name:_____________________________Signature:__________________Date: ______

**Instruction**: For the questions that have alternatives, encircle to the response of the mother and write appropriate response (s) on the space provided for questions for which alternatives are not given.

**Thank you in advance for your cooperation!**

Part I: Individual and community level predisposing characteristic influencing health service seeking of pregnant women for minimum number of ANC visits in Gida Ayana Woreda, rural western Ethiopia.

| S/N | Questions | Response option | remark |
| --- | --- | --- | --- |
| 10 | Women’s age at last pregnancy | Age in complete years [________________] |  |
| 11 | What is your marital status? | Single-------------------------------------0  Married ----------------------------------1  Divorced---------------------------------2  Widowed---------------------------------3  Others [specify] ------------------------99 |  |
| 12 | What is your religion? | Protestant---------------------------------0  Orthodox---------------------------------1  Muslim-----------------------------------2  Catholic----------------------------------3  Other [Specify] ------------------------99 |  |
| 13 | What is your ethnicity? | Oromo------------------------------------0  Amhara ----------------------------------1  Tigire-------------------------------------2  Other [Specify]-------------------------99 |  |
| 14 | The number of children was /were born to a woman? | Number [__________________________] |  |
| 15 | Pregnant women’s educational status? | Cannot read and write -------------------0  Able to read and write -------------------1  Primary school [1-8] ---------------------2  Secondary school [10-12] ---------------3  College diploma or higher -------------4 |  |
| 16 | Husband’s educational status? | Cannot read and write -------------------0  Able to read and write -------------------1  Primary school [1-8] ---------------------2  Secondary school [10-12] ---------------3  College diploma or higher -------------4 |  |
| 17 | Decision on family resources for visiting ANC clinics | Husband alone------------------------------1  Wife/Husband------------------------------2  Wife alone----------------------------------3 |  |

Part II: Enabling behavioral characteristic influencing pregnant women to use decentralized health facilities for a minimum number of ANC.

| **S/N** | **Question** | **Response** | **Remark** |
| --- | --- | --- | --- |
| 18 | What is maternal occupation during last pregnancy? | Housewife----------------------------------0  Government Employed. -------------------1  Small business/service---------------------2  Farmer---------------------------------------  If other [specify]--------------------------99 |  |
| 19 | What is your husband’s occupation during last pregnancy? | Farmer--------------------------------------1  Merchant-----------------------------------2  public employee/private employee-----3  If other [specify]--------------------------99 |  |
| 20 | Estimated household monthly income? | In Ethiopian birr: [_________________] |  |
| 21 | Estimated walking time to nearby decentralized healthcare facilities for minimum number of ANC visits? | In minutes: [______________________] |  |
| 22 | Frequency of home visits by HEWs during the last pregnancy period | No of visits made: [________________] |  |
| 23 | Presence of functional radio and /or TV | Yes-------------------------------------------0  No--------------------------------------------1 |  |
|  |  |  |  |
| 303 | Location of maternal residence? | Urban----------------------------------------0  Rural-----------------------------------------1 |  |
| 304 | Maternal *Kebele* | Write name your *kebele* [____________] |  |
| 304 |  |  |  |
| 305 | How did you judge the availability of motorized transport service during your last labor? | Easily available ----------------------------0  Difficult--------------------------------------1 |  |
| 306 | Decentralized health facility available nearby your home during last birth? | Hospital or clinic---------------------------0  Health center--------------------------------1  Health post----------------------------------2  If other [specify]-------------- -----------99 |  |
| 307 | Place of delivery for last pregnancy? | Hospital or clinic---------------------------0  Health center--------------------------------1  Health post----------------------------------2  Home----------------------------------------3 |  |

Part III. Perceived maternal danger sign complication characteristics influencing the use of decentralized health care facilities for minimum number of ANC visits.

| **S/N** | **Question** | **Response** | **Remark** |
| --- | --- | --- | --- |
| 24 | Have you faced severe headache during your last pregnancy? | Yes----------------------------------------0  No-----------------------------------------1 |  |
| 25 | Have you faced vaginal bleeding/gush during your last pregnancy? | Yes----------------------------------------0  No-----------------------------------------1 |  |
| 26 | Have you faced swelling of hands/face during your last pregnancy? | Yes----------------------------------------0  No-----------------------------------------1 |  |
| 27 | Have you faced severe high fever during your last pregnancy? | Yes----------------------------------------0  No-----------------------------------------1 |  |
| 28 | Have you faced severe sever abdominal pain during your last pregnancy? | Yes----------------------------------------0  No-----------------------------------------1 |  |
| 29 | Have you faced blood pressure during your last pregnancy? | Yes----------------------------------------0  No-----------------------------------------1 |  |
| 30 | Have you faced blurred vision during your last pregnancy? | Yes----------------------------------------0  No-----------------------------------------1 |  |

Part IV: External environmental characteristic influencing visits to local health facilities for ANC.

| **S/N** | **Question** | **Response** | **Remark** |
| --- | --- | --- | --- |
| 31 | Decentralized administrative entity of woman during her last pregnancy? | Ayana----------------------------------------1  Ejere------------------------------------------2  Angar-----------------------------------------3  Lalistu----------------------------------------4 |  |
| 32 | Woman’s residential location during last pregnancy? | Urban----------------------------------------0  Rural-----------------------------------------1 |  |

Part V: Characteristics of ANC utilization and local health facilities attended

| **S/N** | **Question** | **Response** | **Remark** |
| --- | --- | --- | --- |
| 33 | Number of visit(s) ANC woman have made to clinics during last pregnancy? | No visit---------------------------------------------1  One visit--------------------------------------------2  Two visit -------------------------------------------3  Three visit -----------------------------------------4  Four visit -------------------------------------------5  Five visit -------------------------------------------6  Six visit or mor------------------------------------7 |  |
| 34 | Decentralized healthcare facility types a woman visited to obtain a minimum ANC during her last pregnancy? | Hospital/clinic-------------------------------------1  Health center--------------------------------------2  Health post-----------------------------------------3 |  |
| 35 | Decentralized administrative *kebele* by ANC visits | Ayana-----------------------------------------------1  Ejere------------------------------------------------2  Angar-----------------------------------------------3  Lalistu----------------------------------------------4  No visit --------------------------------------------5 |  |
| 36 | Timing of 1^st^ ANC visit? | 1st trimester---------------------------------------1  2nd trimester--------------------------------------2  3rd or 4th trimesters-----------------------------3  4^th^ or more trimesters----------------------------4  No visit--------------------------------------------5 |  |

Part VI: Reasons for woman’s non-utilization of local health facilities for ANC service

| **S/N** | **Question** | **Response** | **Remark** |
| --- | --- | --- | --- |
| 37 | Lack of awareness | Yes----------------------------------------1  No-----------------------------------------2 |  |
| 38 | Transportation problem | Yes----------------------------------------1  No-----------------------------------------2 |  |
| 39 | Waiting time | Yes----------------------------------------1  No-----------------------------------------2 |  |
| 40 | Illness was not severe | Yes----------------------------------------1  No-----------------------------------------2 |  |
| 41 | Heavy workload | Yes----------------------------------------1  No-----------------------------------------2 |  |
| 42 | Others | Yes----------------------------------------1  No-----------------------------------------2 |  |

**...................................END......................................................**

**Thank you once again!**
