# Supplementary material for: Using Andersen’s behavioral model of health care utilization in a decentralized program to examine the use of antenatal care in rural western Ethiopia
Source: PLoS One. 2020 Jan 27;15(1):e0228282. doi: 10.1371/journal.pone.0228282 (PMC6984696; doi:10.1371/journal.pone.0228282)
Supplement: Supplementary file 2 [file pone.0228282.s001.docx]

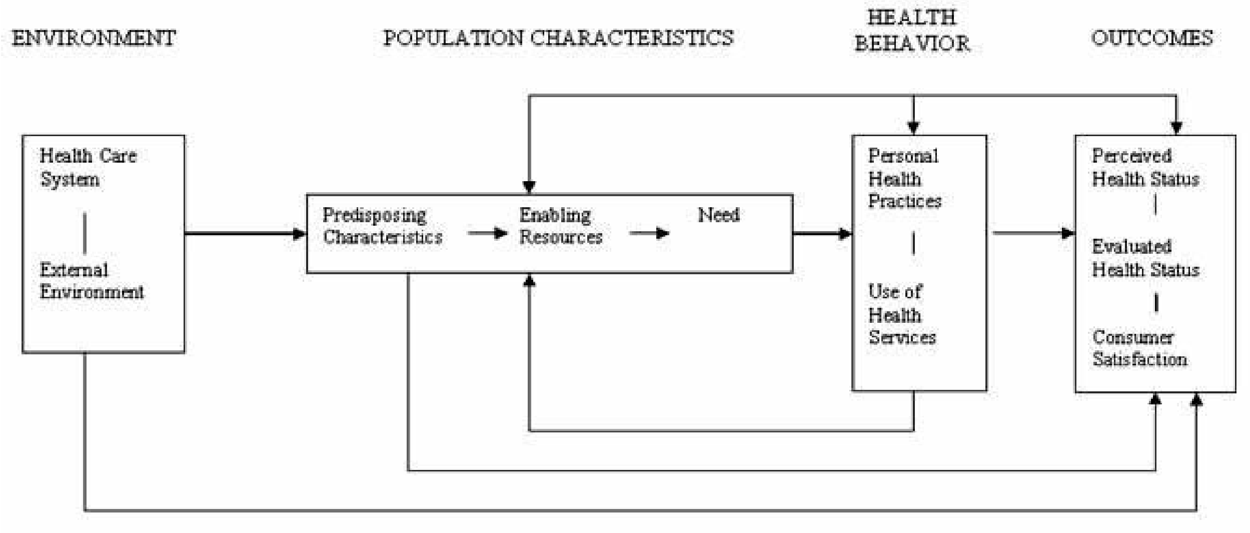
ENVIRONMENT
POPULATION CHARACTERISTICS
HEALTH BEHAVIOR
OUTCOMES
Health Care System
External Environment
Predisposing Characteristics
Enabling Resources
Need
Personal Health Practices
Use of Health Services
Perceived Health Status
Evaluated Health Status
Consumer Satisfaction
