# Supplementary material for: Using Andersen’s behavioral model of health care utilization in a decentralized program to examine the use of antenatal care in rural western Ethiopia
Source: PLoS One. 2020 Jan 27;15(1):e0228282. doi: 10.1371/journal.pone.0228282 (PMC6984696; doi:10.1371/journal.pone.0228282)

PLOS ONE

Using Andersen's behavioral model of health care utilization in a decentralized program to examine the use of ANC in rural western Ethiopia

--Manuscript Draft--

**Manuscript Number:**	PONE-D-19-25468
**Article Type:**	Research Article
**Full Title:**	Using Andersen's behavioral model of health care utilization in a decentralized program to examine the use of ANC in rural western Ethiopia
**Short Title:**	Andersen's behavioral model of health care utilization and the use of antenatal care services
**Corresponding Author:**	Habtamu Tolera<br>Addis Ababa University<br>Addis Ababa, Oromia ETHIOPIA
**Keywords:**	decentralized health services; recommended ANC visits; Andersen model; Gida Ayana Woreda; rural western Ethiopia.
**Abstract:**	Abstract<br>Background: In Ethiopia , most women do not make the minimum number of antenatal care (ANC) visits recommended by WHO. This study modeled predisposing, enabling, need, and external environmental factors in the utilization of decentralized health facilities for ANC services in rural western Ethiopian communities.<br>Methods and findings : A community-based, cross-sectional design was used to study 454 ANC clients in Gida Ayana Woreda (District). Andersen's model of health services-seeking behavior guided the selection of study variables. Multinomial logistic regression was used to model the association between the explanatory variables and the use of recommended and fewer than recommended visits for ANC with reference to the base model, no ANC visits. Adherence to the recommended number of ANC service visits was a function of predisposing, enabling, need, and external environmental factors. Women who made the recommended number of ANC visits constituted 15.2% of all subjects. Christian women made fewer than the recommended ANC visits by 30.0% (Adjusted Odd Ratio, or AOR, = 3.3, $p < 0.01$). Delivery of 2 or more and 1-time home visits by health extension workers (HEWs) was associated with the recommended number of ANC visits. Women with fewer than 2 children (AOR = 10.7, $p < 0.01$) or 2 to 3 children (AOR = 5.5, $p < 0.01$) made the recommended number of ANC visits compared to those with 4 children or more . Women's involvement in gainful activities had higher log odds of seeking the recommended ANC services compared to housewives. Women who experienced high fever were more likely to obtain the recommended ANC services (AOR = 7.1, $p < 0.01$). Residents of Ayana Kebele were 60% more likely to make the recommended number of ANC visits than those in Ejere Kebele . Urban residents were more likely to make the recommended number of visits to ANC clinics (AOR = 2.1, $p < 0.05$).<br>Conclusions : Utilization of recommended ANC services was strongly linked with predisposing, enabling, need, and external factors. This finding indicates the need for an ANC program intervention aimed at meeting WHO recommendations for the minimum number of ANC visits in rural western Ethiopia.
**Order of Authors:**	Habtamu Tolera
	Tegegne Gebre-Egziabher
	Helmut Kloos
**Additional Information:**	
**Question**	**Response**
**Financial Disclosure**<br><br>Enter a financial disclosure statement that describes the sources of funding for the	Habtamu Tolera was the author who received the grant.The research was financially supported by Department of Geography and Environmental Studies of Addis Ababa University and Wollega University. The funders had no grant numbers. The funders had no responsibility in the study design, data collection, analysis and interpretation,

Powered by Editorial Manager® and ProduXion Manager® from Aries Systems Corporation

work included in this submission. Review the submission guidelines for detailed requirements. View published research articles from *PLOS ONE* for specific examples.<br><br>This statement is required for submission and **will appear in the published article** if the submission is accepted. Please make sure it is accurate.<br><br>**Unfunded studies**<br>Enter: *The author(s) received no specific funding for this work.*<br><br>**Funded studies**<br>Enter a statement with the following details:<br>• Initials of the authors who received each award<br>• Grant numbers awarded to each author<br>• The full name of each funder<br>• URL of each funder website<br>• Did the sponsors or funders play any role in the study design, data collection and analysis, decision to publish, or preparation of the manuscript?<br>• **NO** - Include this sentence at the end of your statement: *The funders had no role in study design, data collection and analysis, decision to publish, or preparation of the manuscript.*<br>• **YES** - Specify the role(s) played.<br><br>* typeset	and preparation of the manuscript for publication.
**Competing Interests**<br><br>Use the instructions below to enter a competing interest statement for this submission. On behalf of all authors, disclose any competing interests that could be perceived to bias this work—acknowledging all financial support and any other relevant financial or non-financial competing interests.<br><br>This statement **will appear in the published article** if the submission is accepted. Please make sure it is accurate. View published research articles from *PLOS ONE* for specific examples.	he authors have declared that no competing interests exist.

NO authors have competing interests

Enter: *The authors have declared that no competing interests exist.*

Authors with competing interests

Enter competing interest details beginning with this statement:

I have read the journal's policy and the authors of this manuscript have the following competing interests: [insert competing interests here]

* typeset

Ethics Statement

Enter an ethics statement for this submission. This statement is required if the study involved:

- Human participants
- Human specimens or tissue
- Vertebrate animals or cephalopods
- Vertebrate embryos or tissues
- Field research

Write "N/A" if the submission does not require an ethics statement.

General guidance is provided below. Consult the submission guidelines for detailed instructions. **Make sure that all information entered here is included in the Methods section of the manuscript.**

Research ethics and consent

The research protocol was reviewed and approved by Wollega University Research Ethics Approval Committee [Ref/No.WU–99529/H1-3/24/11/2016]. Permission was received from Gida Ayana Woreda Health Office. The purpose of the study was explained to all participants and a consent form approved by the Review Board was given to participants. Parents or legal guardians provided consent for all participants under age 18. We emphasized that participation was completely voluntary and that they had the right to withdraw any time during the interview without giving any reason. Confidentiality and anonymity were explained to all participants. We ensured that all participants understood the information given by asking them. The consent form was read aloud for women who could not read or write. Literate women were asked to read the consent form. A written consent in the form of a signature or a thumbprint was obtained from all of the participants. ~~None of the participants refused to be interviewed.~~ Five women wanted to end the interviews early due to personal appointments they had to attend to; they were reported as non-responders.

**Format for specific study types**<br><br>**Human Subject Research (involving human participants and/or tissue)**<br>• Give the name of the institutional review board or ethics committee that approved the study<br>• Include the approval number and/or a statement indicating approval of this research<br>• Indicate the form of consent obtained (written/oral) or the reason that consent was not obtained (e.g. the data were analyzed anonymously)<br><br>**Animal Research (involving vertebrate animals, embryos or tissues)**<br>• Provide the name of the Institutional Animal Care and Use Committee (IACUC) or other relevant ethics board that reviewed the study protocol, and indicate whether they approved this research or granted a formal waiver of ethical approval<br>• Include an approval number if one was obtained<br>• If the study involved *non-human primates*, add *additional details* about animal welfare and steps taken to ameliorate suffering<br>• If anesthesia, euthanasia, or any kind of animal sacrifice is part of the study, include briefly which substances and/or methods were applied<br><br>**Field Research**<br><br>Include the following details if this study involves the collection of plant, animal, or other materials from a natural setting:<br>• Field permit number<br>• Name of the institution or relevant body that granted permission	
**Data Availability**<br><br>Authors are required to make all data underlying the findings described fully available, without restriction, and from the time of publication. PLOS allows rare exceptions to address legal and ethical concerns. See the PLOS Data Policy and FAQ for detailed information.	Yes - all data are fully available without restriction

A Data Availability Statement describing where the data can be found is required at submission. Your answers to this question constitute the Data Availability Statement and **will be published in the article**, if accepted.<br><br>**Important:** Stating 'data available on request from the author' is not sufficient. If your data are only available upon request, select 'No' for the first question and explain your exceptional situation in the text box.<br><br>Do the authors confirm that all data underlying the findings described in their manuscript are fully available without restriction?	
**Describe where the data may be found in full sentences. If you are copying our sample text, replace any instances of XXX with the appropriate details.**<br><br>• If the data are **held or will be held in a public repository**, include URLs, accession numbers or DOIs. If this information will only be available after acceptance, indicate this by ticking the box below. For example: *All XXX files are available from the XXX database (accession number(s) XXX, XXX.).*<br>• If the data are all contained **within the manuscript and/or Supporting Information files**, enter the following: *All relevant data are within the manuscript and its Supporting Information files.*<br>• If neither of these applies but you are able to provide **details of access elsewhere**, with or without limitations, please do so. For example:<br><br>*Data cannot be shared publicly because of [XXX]. Data are available from the XXX Institutional Data Access / Ethics Committee (contact via XXX) for researchers who meet the criteria for access to confidential data.*<br><br>*The data underlying the results presented in the study are available from (include the name of the third party*	ll relevant data are within the manuscript and its Supporting Information files.

and contact information or URL).<br>• This text is appropriate if the data are owned by a third party and authors do not have permission to share the data.<br><br>* typeset	
Additional data availability information:	Tick here if the URLs/accession numbers/DOIs will be available only after acceptance of the manuscript for publication so that we can ensure their inclusion before publication.

Powered by Editorial Manager® and ProduXion Manager® from Aries Systems Corporation

Main document Click here to access/download;Manuscript;Manuscript.doc

Using Andersen's behavioral model of health care utilization in a decentralized program to examine the use of ANC in rural western Ethiopia

Habtamu Tolera*[12], Tegegne Gebre-Egziabher[1], Helmut Kloos[3]

[1]Department of Geography and Environmental Studies, Addis Ababa University, Addis Ababa, Ethiopia. [2]Department of Geography and Environmental Studies, Wollega University, Nekemete, Ethiopia. [3]Department of Epidemiology and Biostatistics, University of California, San Francisco, USA.

Corresponding author: *habtol@yahoo.com

Abstract

Background: In Ethiopia**,** most women do not make the minimum number of antenatal care (ANC) visits recommended by WHO. This study modeled predisposing, enabling, need, and external environmental factors in the utilization of decentralized health facilities for ANC services in rural western Ethiopian communities.

Methods ~~and findings:~~ A community-based, cross-sectional design was used to study 454 ANC clients in Gida Ayana *Woreda* (District). Andersen's model of health services-seeking behavior guided the selection of study variables. Multinomial logistic regression was used to model the association between the explanatory variables and the use of recommended and fewer than recommended visits for ANC with reference to the base model, no ANC visits. Adherence to the recommended number of ANC service visits was a function of predisposing, enabling, need, and external environmental factors. Women who made the recommended number of ANC visits constituted 15.2% of all subjects. Christian women made fewer than the recommended ANC visits by 30.0% (Adjusted Odd Ratio, or AOR, = 3.3, $p < 0.01$). Delivery of 2 or more and 1-time home visits by health extension workers (HEWs) was associated with the recommended number of ANC visits. Women with fewer than 2 children (AOR = 10.7, $p < 0.01$) or 2 to 3 children (AOR = 5.5, $p < 0.01$) made the recommended number of ANC visits compared to those with 4 children or more**.** Women's involvement in gainful activities had higher log odds of seeking the recommended ANC services compared to housewives. Women who experienced high fever were more likely to obtain the recommended ANC services (AOR = 7.1, $p < 0.01$). Residents of Ayana *Kebele* were 60% more likely to make the recommended number of ANC visits than those in Ejere *Kebele*. Urban residents were more likely to make the recommended number of visits to ANC clinics (AOR = 2.1, $p < 0.05$).

Conclusions: Utilization of recommended ANC services was strongly linked with predisposing, enabling, need, and external factors. This finding indicates the need for an ANC program intervention aimed at meeting WHO recommendations for the minimum number of ANC visits in rural western Ethiopia.

~~**Keywords:** decentralized health services; recommended ANC visits; Andersen model; Gida Ayana Woreda; rural western Ethiopia~~

Introduction

About 300,000 maternal deaths were reported worldwide in 2013. Of these, 99% were in the developing world, making the maternal mortality rate in that region 230 per 100,000 live births, which was 14 times higher than in industrialized countries. Sub-Saharan African women accounted for 62% of the global maternal deaths and had the highest maternal mortality rate, 510 deaths per 100,000 live births in 2013.[1]

In Ethiopia, 15,234 maternal deaths were reported in 2013.[2–4] The Ethiopian Demographic Health Surveys of 2005 and 2011 revealed maternal death ratios of 673 and 676 per 100,000 live births, respectively, showing no change between the two studies.[5,6] ~~The Health Sector Development Program (HSDP) IV of the country that was implemented in 2010 set a target maternal death rate of 267 deaths per 100,000 live births by 2015.[7] Achieving this goal would enable the country to meet the Millennium Development Goal5 (MDG5) of 350 maternal deaths per 100,000 live births.[2,7]~~ However, the countrywide 2016 Demographic Health Survey documented a maternal death rate of 412, far short of the HSDP IV and MDG5 targets.[8–10] Most maternal deaths (90%) are avoidable with timely interventions[11,12] The prevention of maternal mortality is a priority for the Ethiopian government and the World Health Organization; the UN Sustainable Development Goal3 calls for reducing the global maternal mortality ratio to less than 70 per 100,000 live births by 2030.[8,13]

Studies carried out elsewhere have found that simple ANC interventions such as monitoring blood pressure and body weight, giving vaccinations, and providing counseling on pregnancy and danger signs are highly effective preventive measures.[11,14–17] Non-rutilization of local ANC programs may help explain the persisting high rates of pregnancy complications in Ethiopia.[14,18,19] Despite the Ethiopian government's efforts to improve maternal health and bring

facilities closer to mothers through decentralization programs implemented in the early 1990s.[20,21,22], ANC utilization remains low.[3,23,24] The 2016 Demographic Health Survey, which covered all the regions of Ethiopia, found that 49.3% of women in the most populous administrative region, Oromia, did not receive ANC, and results were similar for Somali Region.[9] This statistics suggests a gap in understanding of the impact of multifaceted factors on ANC utilization, particularly in Oromia Region, where antenatal outcomes are poor compared to national figures.[8] Preventable maternal health risks may be managed with early detection.[11,15,18,25,26] This study examined antenatal service utilization behavior of women in a remote rural area in western Ethiopia**.**

Various studies have reported that antenatal healthcare service utilization is affected by a number of factors including individual attitudes and health-care-seeking behaviors; household income; socio-cultural practices; and promotion and awareness of antenatal service packages among service users, religious leaders, the wider communities, and other stakeholders.[18,27–30] However, the determinants of utilization of antenatal health care services vary across different geographical locations and contexts, different cultures and beliefs, and different socioeconomic and demographic settings.[15,17,24,29,31,32]

There is little information about women’s utilization of decentralized ANC services and underlying factors in Oromo culture or the impact of the historical dominance of Christian missionary health services in rural western Ethiopia. Oromia Region has its own peculiar geographic, socioeconomic, and cultural characteristics that may influence the utilization of ANC services. ~~This study is based on Andersen’s model of health care utilization.[30,33] We used this model because it was helpful in identifying predisposing, enabling, need, and external environmental factors in ANC services utilization and informing health decision making.~~ In

order to understand the uptake of ANC, ANC outcomes need to be modeled as functions of predisposing, enabling, perceived need, and environmental factors.[18,26,34,35] Andersen developed his model to ascertain conditions that facilitate or impede health-seeking behavior of individuals.[18,26,35] The model is a robust analytical tool and can be widely applied in different settings.[29,30,33]

Studies employing Andersen's model in studying maternal health in Ethiopia have used secondary sources; thus there is a need for studies using primary data.[3,27,35] Hence, the objective of this study was to investigate the above domains of determinants influencing the use of decentralized health facilities for the recommended number of ANC services with the aim of informing policy makers and practitioners responsible for planning, administering, and delivering maternal health service programs.

Methodology

Study setting

The study was conducted in Gida Ayana *Woreda*, Oromia Region, western Ethiopia, about 450 km from Addis Ababa, and 112 km from Nekemte, the capital of Eastern Wollega Zone. The area of the *woreda* is about 1,502 square km and has 21 rural and 7 urban *kebeles* (the smallest administrative units in Ethiopia). According to the 2013 population projection release and Eastern Wollega Zone, the *woreda* had a total population of 140,484, including 47,040 child-bearing women and 10, 577 women of reproductive age (15–49 years).[36,37]

Study design and period

A population-based, cross-sectional design was used in this study, conducted from November 2016 to January 2017.

Sample and recruitment

The sample size was determined using a single population proportion formula.[38] Following a previous study,[39] a proportion of 32.7%, a 95% confidence interval (CI), a margin of error of 5%, and a design effect of 1.5 were used. Thus, a minimum adequate sample size was determined using the statistical estimation method.[38] Since the source population was assumed to be less than 10,000, the sample size was corrected by adding 5% for non-responses, giving a final sample size of 459 women.

Two-stage sampling using a simple random sampling technique was employed to select an appropriate representative study population. In the first stage, four representative *kebeles* (Ayana, Angar, Ejere, and Lalistu) were randomly selected using the lottery technique. In the four selected *kebeles*, households having women who reported to have had their last birth during the 5 years prior to the study were identified with the help of female health extension workers and women team leaders. In the second stage, eligible women were sampled using Microsoft Office Excel-generated random numbers proportional to the estimated number of women who had given birth in the respective *kebeles* during those 5 years. When two women were living in the same household, recent births were considered in determining whom to interview. Where study mothers were not available during the survey, they were visited again the next day and, if not available then, they were considered to be non-responders. Inclusion criteria of our study were women aged 15–49 years who gave birth to children during the 5 years prior to the survey. Women who were ill were excluded from the study because they were considered unable to provide reliable information on their pregnancy care.

Outcome

ANC is defined here as at least one visit to a doctor, nurse, midwife, or trained traditional birth attendant during pregnancy.[40] The nominal dependent variable of the study was the number of visits to ANC service clinics. ANC users were categorized into three groups according to the WHO recommendations for ANC visits, irrespective of when in the course of the pregnancy the visits occurred; the groups were as follows: the logit or the log-odds of having y = 2 [those who made the recommended 4 or more ANC visits], y = 1 [those who made 1 to 3 ANC visits, fewer than the recommended number]; and y = 0 [no ANC visits]. No ANC visit was a base model for the first two modeled categories.

Explanatory variables

Andersen's behavioral model of health care utilization, which provides the theoretical framework for our study, posits that the decision to seek services is influenced by both context and individual behavior-specific factors.[17, 18, 29, 30, 33] (Fig 1). The model identifies three groups of internal factors that determine people's use of health services: (a) a person's predisposition to seek health services, (b) factors that enable or prevent a person's use of the service, and (c) a person's perceived need for the services. Predisposing factors include individual characteristics that are not directly related to health but influence the likelihood of utilization of health services. Enabling factors are means individuals have or do not have at their disposal for accessing the services. The need factors arise from an individual's health status. Based on this theory, we identified and examined the following determinants of adherence to the recommended number of visits to ANC clinics. As predisposing factors, we selected age as a three-categorical variable (19 or less, 20-34, 35-48 years), mother's and father's education (college or higher, secondary,

primary, no education), marital status (married or other), mother's religion (Christian or Moslem), and decision making regarding use of household resources (husband only, husband/wife, wife only). Enabling factors were mother's employment (skilled employee, small business/service, farmer, housewife), husband's employment (skilled employee, merchant, farmer, other), home visits by health extension workers [HEWs] (more than one, one, none), distance to ANC (under 30 minutes, 30 minutes or more), household income (less than 50 $US, 50 $US or more), and possession of a radio/TV (yes or no). Need factors were severe headache, vaginal bleeding/gush, swelling of hands/face, high fever, severe pain in the abdomen, high blood pressure, and blurred vision, each classified as a binary variable (yes or no). As external environmental factors, we examined the administrative *kebeles* where the women lived and in which health facilities were decentralized as well as whether the women lived in urban or rural locations. Using these external factors enabled us to explore differences in ANC utilization across space and to consider the available decentralized health facility type (health post, health center, hospital/clinic) as an enabling factor.

Fig. 1 Conceptual framework of the study

Data collection and quality control

Data were collected using a structured questionnaire developed ~~according to the objective of the study and the~~ review of the literature. The questionnaire was designed in English and translated to Afan Oromo by staff of the English and Local Language departments of Wollega University. Data collection focused on institutional delivery; place of delivery; and predisposing, enabling, and perceived needs factors affecting ANC use. Data were collected by 8 ~~health extension workers (~~HEWs~~)~~ with BSc degrees or diplomas in health science who had previous experience in data collection in Gida Ayana *Woreda*. Data collection was supervised by two supervisors

recruited from a local health center and coordinated by the corresponding author. The data collectors and supervisors were trained for two days in data collection techniques and ethics. The questionnaire was pretested for construct validity with a 10% sample in a nearby *woreda* and modified. Each completed questionnaire was checked daily to ascertain that all the questions were correctly answered to address data validity and reliability.

Data processing and analysis

We used statistical software EpiData ~~Entry~~ Version 3.1 for preliminary data preparation and statistical software SPSS Version 24.0 for data analysis. Means and standard deviation and cross tab were used to measure continuous and categorical variables, respectively. Another statistic of interest is the Variance Inflation Factor (VIF)~~. A VIF~~ > 10 indicates redundancy among explanatory variables.[41,42] Our ANC utilization model satisfied this criterion with VIF < 2.0. Associations between the number of ANC service visits in the three groups (those receiving the recommended number of ANC visits, those receiving fewer than recommended, and those receiving no ANC) and explanatory variables were calculated by the use of the binomial and multinomial logit (MNL) model. Those explanatory factors that displayed statistical significance ($p < 0.05$) in the bivariate analyses were entered into the final multinomial regression model. The adequacy of the developed model was verified through the standard statistical mean of likelihood ratio test of goodness of fit.[42] Multicollinearity in the MNL model was detected by examining the standard error for the coefficients.[16] The association of variables was found to be statistically significant at $p < 0.05$. Adjusted odds ratios (AOR) with corresponding 95% CI estimates were used to describe the strength of associations of factors with recommended number of ANC visits and fewer than the recommended number of ANC visits versus no visits.

Research ethics and consent

The research protocol was reviewed and approved by Wollega University Research Ethics Approval Committee [Ref/No.WU–99529/H1-3/24/11/2016]. Permission was received from Gida Ayana *Woreda* Health Office. The purpose of the study was explained to all participants and a consent form approved by the Review Board was given to participants. Parents or legal guardians provided consent for all participants under age 18. We emphasized that participation was completely voluntary and that they had the right to withdraw any time during the interview without giving any reason. Confidentiality and anonymity were explained to all participants. We ensured that all participants understood the information given by asking them. The consent form was read aloud for women who could not read or write. Literate women were asked to read the consent form. A written consent in the form of a signature or a thumbprint was obtained from all of the participants. None of the participants refused to be interviewed. Five women wanted to end the interviews early due to personal appointments they had to attend to; they were reported as non-responders.

Results

Socio-demographic background

A total of 459 women who had their last birth during the 5 years preceding the survey were enrolled in the study. The response rate was 98.9%. The mean age of mothers was 26.1 (±7.1) years. The mean number of children women gave birth to was 3.11 (±2.0). Nearly half of the mothers reportedly had no formal education. Over one-third of the husbands had completed secondary education and 33.9% completed college/higher education. Almost half of the respondents were of the Oromo ethnic group, 83.9% were married, and 55.9% were urban. Over

half of mothers were Christian. One hundred ninety-eight (43.6%) were housewives and 124 (27.5%) were paid employees. Most husbands were subsistence farmers. Mean walking time to the nearest health facility was 51.1(±31) minutes, and the mean monthly household income was 47 (±15.1) $US. (**Table 1**).

Table 1. Socio-~~cultural and~~ demographic backgrounds of the study participants (*N = 454*), Gida Ayana *Woreda*

Variable	Variable ~~level~~	Number(*n*)	Percentage (%)
Age (in years**)**	[illegible] or less	127	28.0
	20-34	255	56.1
	35 or more	72	15.9
Education	No formal education	224	49.3
	Primary	81	17.8
	Secondary	73	16.1
	College/higher	76	16.7
Husband's education	No formal education	90	19.8
	Primary	49	10.8
	Secondary	161	35.5
	College/higher	154	33.9
Occupation	Paid employee	124	27.5
	Small business/service	98	21.6
	Farmer	34	7.3
	Housewife	198	43.6
Husband's occupation	~~Skilled~~ employee	86	18.9
	Merchant	114	25.1
	[a]Informal activity	42	9.3

	Farmer	212	46.7
Marital status	Married	381	83.9
	[b]Other	73	16.1
Ethnicity	Oromo	222	48.9
	Amhara	144	31.7
	Tigre	88	19.4
Religion	Christian	260	57.3
	Moslem	194	42.7
Home visit by HEWs	2 times or more	132	29.0
	One time	67	14.8
	No	255	56.2
Urban-rural location of the women	Urban	254	55.9
	Rural	200	44.1
Mean number of births (SD)	3.11(±1.9)		
Mean walking time to ANC clinic in min (SD)	51.1(±30.9)		
Mean monthly family income in US$ (SD)	47.0(±15.1)		

[a]Informal activity: day laborer/weaving/studen[illegible]ther: single/widowed/divorced. SD: Standard Deviation.

ANC service use and decentralized facility attended

The majority (55.1%) of the women obtained ANC from a decentralized health center facility. The women who made the recommended number of visits to ANC constituted 15.2% of the participants while 49.6% made fewer than the recommended number and 35.2% did not seek services. We found that 89.4% and 66.2% of respondents of Ayana and Ejere *Kebeles*, respectively, visited the ANC clinic, and lower proportions did so in Angar and Lalistu *Kebeles*. Of the women who utilized ANC services, 41.5% made their first visit in the second trimester of their pregnancy. Among those who made no ANC visit, 46.25%, 25.0%, and 11.25% mentioned

lack of awareness about the importance of pregnancy care, transportation problems, and long waiting times, respectively, as reasons for not using ANC services (**Table 2**).

Table 2 Utilization of ANC and local health facilities attended, Gida Ayana *Woreda*

Variable	Variable level	Number(n)	Percentage(%)
ANC visits	Recommended # ANC visits	69	15.2
	Fewer than recommended visits	225	49.6
	No visits	160	35.2
Decentralized facility visited	Hospital/clinic	25	8.5
	Health center	162	55.1
	Health post	107	36.4
Decentralized administrative *kebele* by ANC visits	Ayana	84	89.4
	Ejere	51	66.2
	Angar	92	57.5
	Lalistu	67	54.5
Timing of 1st ANC visit	1st trimester	96	32.6
	2nd trimester	122	41.5
	3rd or 4th trimesters	76	25.9
[c]Reason for no ANC visit	Lack of awareness	74	46.25
	Transportation problem	40	25.0
	Waiting time	18	11.25
	Illness was not severe	14	8.75
	Heavy workload	10	6.25
	Others	4	2.5
	Total	160	100

[c]multiple responses were possible.

Factors influencing visits to ANC in a decentralized facility

Table 3 shows the bivariate association between explanatory variables and number of visits to ANC facilities. All factors except high blood pressure and blurred vision were significantly associated ($p < 0.05$) with the outcome variable. In the following sections, we compare the association between determinant factors and recommended number of ANC visits and fewer than the recommended ANC visits versus no ANC visits as established through the multinomial regression analysis (**Table 4**).

Predisposing factors

Holding other variables constant, Christian women were 3.3 times more likely than Muslims to make 3 or fewer ANC visits than no visits. Nevertheless, identification as Christian was not significantly associated with utilization of the recommended number of ANC visits although the corresponding log odds figure was higher (AOR = $p > 0.279$).

Women who had fewer than 2 children were 10.7 times and 9.2 times more likely to make the recommended number of ANC visits (AOR= 10.7, $p < 0.01$) and fewer than the recommended number (AOR= 9.2, $p < 0.01$), respectively, than no visits compared to women who reported 4 children or more. Likewise, women with 2 to 3 children had log odds of 5.5 times and 4.6 times higher of making the recommended number of ANC visits (AOR= 5.5, $p < 0.05$) and fewer than the recommended ANC visits (AOR= 4.6, $p < 0.01$), respectively, than no visits compared to women who had 4 or more children.

Gendered decision making about resource use influenced women's maternal health service seeking behavior in choosing the recommended number of ANC visits rather than no visits, but the association was not statistically significant. However, when all other factors were held constant, shared decision making (women/husband) about household resource use was 3.9 times

more likely to result in fewer than the recommended ANC clinic visits than no visits compared to wife-only decision making (AOR= 3.9, $p < 0.01$).

Enabling factors

When all other determining factors were held constant, women who operated small businesses had 4.0 times and 2.2 times higher log odds of choosing recommended and fewer than the recommended number of ANC visits, respectively, than no visits compared to women who identified as housewives.

~~Women in households with monthly household income of 50 $US or more were nearly 3 times and 2 times more likely to make the recommended (AOR=2.8, $p < 0\ 05$) and fewer than recommended number of ANC visits (AOR=2.1, $p < 0.05$), respectively, than no visits compared to those reporting less than 50 $US in household income.~~

~~Women who were visited 2 times or more than 2 times by HEWs were 9.7 times and 4.2 times, respectively, more likely to make the recommended (AOR = 9.7, $p < 0.05$) and fewer than recommended number of ANC visits (AOR = 4.2, $p < 0.05$) than no visits compared to those reporting no visits by HEWs. Likewise, women who were visited one time had higher log odds of making the recommended (AOR = 9.5, $p < 0.01$) and fewer than recommended (AOR = 4.8, $p < 0.01$) number of ANC visits by 50% and 80%, respectively, than no visits compared to those who were not visited by HEWs.~~

~~Walking time strongly influenced the utilization of health facilities, specifically the number~~ of ~~ANC visits, but the relationship was not statistically significant. Furthermore, the log odds ratio of receiving fewer than 4 ANC visits versus no visits was 40% higher for women living closer than 30 minutes from the nearest ANC clinics (AOR = 2.4, $p < 0.05$).~~

When comparing type of decentralized health facility, no significant association was found with the utilization of the recommended number of ANC visits versus no visits. For women who received services at decentralized front-line health posts, the log odds of making fewer than the recommended number of ANC visits versus no visits to health centers was 20.0% higher (AOR = 2.2, $p < 0.05$).

Need factors

When all other determining factors were held constant, women who reportedly felt severe headaches were found to be 90% (AOR = 2.9, $p < 0.05$) and 70% (AOR = 3.7, $p < 0.01$) more likely to make the recommended and fewer than recommended number of ANC visits, respectively, than those not reporting headaches. The log odds ratio for making fewer than the recommended number of ANC visits was 20% higher (AOR = 2.2, $p < 0.05$) for women who experienced vaginal bleeding/gush compared to those who did not. Likewise, the odds of women who had high fevers were 7.1 and 4.1 times higher for having the recommended (AOR = 7.1, $p < 0.01$) and fewer than recommended (AOR = 4.1, $p < 0.01$) number of visits to ANC facilities, respectively, than those who felt no fever.

External environmental factors

When comparing the utilization of ANC services in the four study *kebeles* in which government decentralized health facilities were located, women of Ayana had higher odds of making the recommended number of visits (AOR = 8.2, $p < 0.01$) and fewer than the recommended number (AOR = 24.6, $p < 0.01$) than making no visits compared to women of Ejere *Kebele*. Furthermore, when all other factors were held constant, residents of urban settlements were 2.1 times more likely to make the recommended number of ANC service visits (AOR = 2.1, $p < 0.01$) compared

to rural residents. However, the urban-rural difference did not seem to significantly influence the choice of fewer than the recommended number of ANC service visits in spite of higher log odds.

Table 3. Bivariate analysis of predisposing, enabling, need, and environmental factors by number of ANC visits

		Status of ANC visits			Number*(n)*	*p*-value
Variable	Categorical level	Recommended	Fewer than recommended	No visit		
Predisposing factors	Age					
	19 or less	21.3	53.5	25.2	127	0.002
	20-34	13.7	51.0	35.3	255	
	35 or more	9.7	37.5	52.8	72	
	Education					
	College or higher	27.6	51.3	21.1	76	0.000
	Secondary	21.9	50.7	27.4	73	
	Primary	12.3	60.5	27.2	81	
	No formal education	9.8	44.6	45.5	224	
	Husband's education					
	College or higher level	28.9	52.2	18.9	90	0.000
	Secondary	18.4	57.1	24.5	49	
	Primary	13.7	51.6	34.8	161	
	No formal education	7.8	43.5	48.7	154	
	Marital status					
	Married	12.9	53.0	34.1	381	0.001
	Other	27.4	31.5	41.1	73	
	Religion					
	Christian	15.8	56.2	28.1	260	0.001
	Moslem	14.4	40.7	44.8	194	

	Number of births					
	Fewer than 2	17.6	59.7	22.7	216	0.000
	2 to 3	16.1	51.0	32.9	149	
	4 or more	7.9	22.5	69.7	89.0	
	Decision on resource use					
	Husband only	19.4	52.5	28.1	139	0.002
	Wife/Husband	11.0	59.8	29.1	127	
	Wife only	14.9	40.4	44.7	188	
Enabling factors	Decentralized facility					
	Hospital/clinics	26.2	33.3	40.5	42	0.000
	Health center	19.4	61.2	19.4	201	
	Health post	9.0	41.7	49.3	211	
	Occupation					
	Paid employee	18.5	50.8	30.6	124	0.000
	Small business	16.3	53.1	30.6	98	
	Farmer	32.4	55.9	11.8	34	
	Housewife	9.6	46.0	44.4	198	
	Monthly income					
	50 $US or more	17.2	53.5	29.3	273	0.004
	Less than 50 $US	12.2	43.6	44.2	181	
	Husband's occupation					
	Skilled employee	26.7	57.0	16.3	86	0.000
	Merchant	16.7	53.5	29.8	114	
	Other	14.3	54.8	31.0	42	
	Farmer	9.9	43.4	46.7	212	
	Visits by HEWs					
	More than one time	22.0	56.8	21.2	132	0.000
	One time	23.9	59.7	16.4	67	
	No visit	9.4	43.1	47.5	24	
	Walking time to ANC clinic					

	29 minutes or less	15.2	53.7	31.1	296	0.028
	30 minutes or more	15.2	41.8	43.0	158	
	Own radio and/or TV					
	Yes	18.5	54.7	26.7	243	0.000
	No	11.4	43.6	45.0	211	
Need factors	Severe headache					
	Yes	12.5	59.0	28.5	144	0.023
	No	16.5	45.2	38.4	310	
	Vaginal bleed/gush					
	Yes	16.1	59.8	24.1	112	0.015
	No	14.9	46.2	38.9	342	
	Swelling hands/faces					
	Yes	18.1	52.8	29.1	265	0.003
	No	11.1	45.0	43.9	189	
	High fever					
	Yes	20.2	61.2	18.6	183	0.000
	No	11.8	41.7	46.5	271	
	Abdominal pain					
	Yes	16.2	52.6	31.2	340	0.007
	No	12.3	40.4	47.4	114	
	High blood pressure					
	Yes	17.8	53.4	28.8	118	0.218
	No	14.3	48.2	37.5	336	
	Blurred vision					
	Yes	13.5	50.6	36.0	178	0.716
	No	16.3	48.9	34.8	276	
External environmental factors	Administrative *kebele* of the women					
	Ayana	30.9	58.5	10.6	94	

	Ejere	14.3	51.9	33.8	77	0.000
	Lalistu	9.8	44.7	45.5	123	
	Angar	10.6	46.9	42.5	160	
	Urban-rural residence					
	Urban	18.1	51.2	30.7	254	0.02
	Rural	11.5	47.5	41.1	200	

[a]The average exchange rate of 1$US was 21.0 Ethiopian Birr between Nov. 2016 and January 2017. *P-values* were from the chi-square test.

Discussion

Although increasing the number of ANC visits has contributed to a drastic reduction in the maternal death rate in low-income countries during the past 30 years, the majority of women in sub-Saharan Africa, including Ethiopia, still do not make the WHO-recommended 4 ANC visits or more during the first trimester.[25] This study found that only 15.2% of the 454 participants received the recommended number of ANC visits; 49.6% made fewer than the recommended ANC visits and 35.2% reported no visits for ANC.

Our findings identified a number of predisposing, enabling, need, and environmental factors influencing the choices regarding ANC visits: religion, number of children, woman's occupation, home visit by HEWs, walking time to health facility, monthly income, headache, vaginal bleeding, fever, availability of decentralized ANC facilities, decentralized administrative *kebele*, and urban-vs-rural residence of the women ~~(Table 4).~~

~~Predisposing factors~~

The results showed that although religion was not significantly associated with making the recommended number of ANC visits, it was associated with the utilization of fewer than the

recommended number of ANC visits. Christian women were almost 3 times more likely to make fewer than the recommended number of ANC visits than no visits compared to Muslim women. This finding is consistent with a study in northeastern Ethiopia, where Christians were 2.2 times more likely to make fewer than the recommended number of ANC visits compared to Muslim women.[43] In Nigeria, Christian women were more likely to make the recommended number of ANC visits than fewer than the recommended number than Muslims.[44] The higher level of ANC use among women of certain religions could be attributed to theological differences and differences in lifestyle across various beliefs.[17] In Nepal, Christians and Hindus were 50% and 30%, respectively, more likely to make the recommended and fewer than recommended number of ANC visits versus no visits compared to women of other religions.[16]

Women experiencing their first pregnancy were 10.7 and 9.2 times more likely to make the recommended and fewer than the recommended number of ANC visits, respectively, than no visits compared to women who had 4 children or more. Similarly, those who had 2 or 3 children were 5.0 and 6.0 times, respectively, more likely to make the recommended and fewer than recommended number of visits than no visits compared to baseline. Recent systematic reviews and meta-analyses reveal that women with first pregnancies are more likely than multiparous women to make the recommended number of ANC visits due to fear of complications with the first birth.[25,26,35] Multiparous women tend to believe there is less risk to home delivery due to their previous birthing experiences and their negative perceptions of the environment in local health institutions regarding cleanliness, equipment quality, and behavior of providers.[18,25,31,45]

Results also showed that women who were able to decide with their partners on family resource use had higher log odds of choosing fewer than the recommended number of ANC visits by 90.0%, which implies that housewives with some autonomy in this area were able to make at

least some visits to clinics. This suggests that women who were not constrained by a patriarchal structure were better able to utilize ANC services. This finding corroborates a study in northern Ethiopia in which decision making by wives and husbands separately was associated with 45% and 65% lower numbers making the recommended and fewer than recommended number of ANC visits, respectively, compared to couples who made the decision jointly.

Table 4 Multinomial regression analysis of predisposing, enabling, perceived need, and environmental factors influencing number of women's ANC visits (*n* = 454).

			Status of ANC visits. Base model: No-visits category			
			Recommended visits		Fewer than recommended visits	
Factor	Variable	Variable level	AOR (95% CI)	*p*-value	AOR (95% CI)	*p-value*
Predisposing factors	Religion	Christian	1.6(0.6-3.7)	0.279	3.3(1.7-6.5)**	0.000
		Moslem	1		1	
	Marital status	Married	0.5(0.1-1.5)	0.253	2.3 (0.9-5.8)	0.06
		others	1		1	
	No. of children	Fewer than 2	10.7(3.0-38.4)**	0.000	9.2(3.6-23.0)**	0.000
		2 to 3	5.5(1.5-20.4)**	0.01	4.6(1.8-11.5)**	0.001
		4 or more	1		1	
	Age	19 or less	1.7(0.4-6.5)	0.402	1.0(0.4-2.7)	0.906
		20-34	0.6(0.2-2.3)	0.557	0.9(0.3-2.1)	0.814
		35 or more	1		1	
	Education	College/higher	1.1(0.2-5.1)	0.902	0.7(0.2-2.7)	0.719
		Secondary	0.8(0.2-2.7)	0.743	0.6(0.2-1.7)	0.395
		Primary	1.7(0.5-6.0)	0.373	2.1(0.8-5.1)	0.092

		No education	1		1	
	Husband's education	College/higher	2.4(0.4-12.7)	0.299	0.8(0.2-3.1)	0.771
		Secondary	1.7(0.3-7.7)	0.468	1.3(0.4-4.1)	0.64
		Primary	1.2(0.4-3.9)	0.662	0.9(0.4-2.0)	0.878
		No education	1		1	
	Decision on family resource	Husband	1.5(0.6-3.9)	0.334	1.9(0.9-4.0)	0.075
		Wife/husband	1.6(0.6-4.6)	0.316	3.9(1.8-8.4)**	0.000
		Wife	1		1	
Enabling factors	Employment	Skilled employee	2.1(0.7-5.8)	0.133	1.4(0.6-2.9)	0.365
		Small business/service	4.0(1.4-11.7)**	0.009	2.2(1.0-4.8)*	0.05
		Farmer	2.3(0.4-13.4)	0.349	1.8(0.3-9.0)	0.424
		Housewife	1			
	Husband's employment	Skilled employee	1.5(0.2-8.1)	0.623	2.0(0.5-7.7)	0.291
		Merchant	1.0(0.3-3.1)	0.972	1.5(0.6-3.5)	0.341
		Other	0.8(0.1-4.0)	0.885	1.3(0.4-3.9)	0.603
		Farmer	1		1	
	Family income	50 $US or more	2.8(1.2-6.2)*	0.011	2.1(1.1-3.8)*	0.013
		Less than 50$US	1		1	
	Home visit by HEWs	More than one time	9.7(3.7-25.2)**	0.000	4.2(1.9-8.9)**	0.000

		One time	9.5(2.9-30.7)**	0.000	4.8(1.8-13.0)**	0.001
		No visit	1		1	
	Walking time to ANC clinic	Less than 30 minutes	1.7(0.7-4.4)	0.226	2.4(1.2-5.0)*	0.013
		30 minutes or more	1		1	
	Possession of radio/or TV	Yes	1.7(0.7-4.1)	0.224	1.7(0.8-3.3)	0.108
		No	1		1	
	Decentralized facilities	Hospital/clinic	1.1(0.2-4.6)	0.889	0.4(0.1-1.6)	0.239
		Health center	2.3(0.9-5.8)	0.08	2.2(1.1-4.5)*	0.019
		Health post	1		1	
Need factors	Severe headache	Yes	2.9(1.1-7.5)*	0.026	3.7(1.8-7.6)**	0.000
		No	1		1	
	Vaginal bleeding/gush	Yes	1.7(0.6-4.6)	0.239	2.2(1.0-4.8)*	0.036
		No	1		1	
	Swelling of hands/face	Yes	1.2(0.5-2.9)	0.601	0.9(0.5-1.8)	0.99
		No	1		1	
	High fever	Yes	7.1(2.9-17.5)**	0.000	4.1(1.9-8.5)**	0.000
		No	1		1	
	Severe pain in abdomen	Yes	0.8(0.3-2.4)	0.794	1.1(0.5-2.5)	0.676

		No	1		1	
External Environmental factors	*Kebele*	Ayana	24.6(4.8-15.2)**	0.000	8.2(2.1-3.5)**	0.002
		Angar	2.0(0.3-11.3)	0.398	0.9(0.2-3.3)	0.951
		Lalistu	1.2(0.3-5.1)	0.742	0.8(0.3-2.5)	0.806
		Ejere	1		1	
	Urban-rural residence	Urban	2.1(1.1-3.7)**	0.013	1.4(0.9-2.1)	0.8
		Rural	1		1	

1 = Reference category; * significant at $p < 0.01$ * **significant at $p < 0.05$

~~Enabling factors~~

~~In this study, the log odds ratios associated with gainful small business activities were 4.0 and 2.2 times higher for making the recommended and fewer than recommended number of ANC visits, respectively. The odds ratios corresponding to the other categories of women's occupation were also higher. However, their *p*-values did not demonstrate statistically significant association with ANC service visits (**Table 4**).~~ This finding is consistent with those of previous studies in Ethiopia.[25,28] A study in Nigeria[46] reported that women who operated small businesses were 6 times more likely to make the recommended number of ANC clinics visits. Similar studies in Nepal and China also corroborate our findings.[16,47]

Furthermore, we found that women in households reporting monthly income of $US 50.00 or more made the recommended and fewer than recommended number of ANC visits at a rate 80% and 10% higher than no visits, respectively, compared to those with household incomes of $US 49 or less. This variation might be an area of concern for policy makers. A study in Sodo

Woreda[25] reported that 13% of rural women with higher cash incomes made the ANC visits recommended by WHO. Similar findings were reported in Nigeria, where women in wealthier households were 4.0 times more likely to make the recommended number of visits.[48] The differentials were 2.71 times in studies in Afghanistan,[49] 8.8 times in Nepal,[16] and 3.3 times in China.[47]

Women who were visited 2 or more times by HEWS were 70% and 20% more likely to make the recommended and fewer than recommended number of visits, respectively, than women who were not visited. Women who were visited only once made the recommended and fewer than recommended number of visits at a 50% and 80%, respectively, higher rate. Similar findings were reported by several other studies.[17,49–51]

Women who visited decentralized health centers were 20% more likely to make 3 or fewer ANC visits than those with utilized nearby bottom-line health posts. This finding corroborates those of other studies, which predicted higher log odds for making at least 3 recommended visits to ANC clinics.[50,52] Women were likely to visit health centers that were better equipped and more user friendly than others.[53] But this result was not consistent with a study in Rwanda, where decentralized health posts were over utilized compared to health centers because health posts were better supplied with maternal resources and attracted most of the local women.[54]

After adjusting for all variables, women were found to be 40% more likely to make 1 to 3 ANC visits to health facilities located within walking distance of half an hour than to facilities located at greater distances. Similar findings have been documented elsewhere.[23,28,55]

Need factors

Women with severe headaches had 9.0 and 6.0 times higher log odds of making the recommended and fewer than recommended number of visits for ANC, respectively; headaches

appeared to motivate mothers to attend ANC clinics. Studies carried out elsewhere support this finding.[23,25,34,45,56] Women reporting high fevers were 7.1 times (AOR = 7.1, $p < 0.01$) and 4.1 times (AOR = 4.1, $p < 0.01$) more likely to make the recommended or fewer than recommended number of visits, respectively. This finding is in line with studies in Hadiya Zone in southern Ethiopia,[57] west Bengal in India,[58] and rural Bangladesh.[45] The reason women with fevers appear motivated to utilize ANC could be that mothers with a history of complications have personal experience that helps them understand the life-threatening condition and makes them inclined to seek preventive maternal care.

External environmental factors

There were geographical variations in the use of ANC among women in the four *kebeles* with government-decentralized health facilities. Recommended and fewer than recommended number of visits to ANC were 60% and 20% higher, respectively, among residents of Ayana *Kebele* compared to residents of Ejer*e Kebele*. ~~The highest log odds ratios for recommended ANC visits were in Angar (AOR = 2.0, $p > 0.05$) and Lalistu (AOR = 1.2, $p > 0.05$),~~ but the figures were not statistically significant. Another study found wide interregional disparities in ANC use in Ethiopia, with Oromia Region having the lowest use of all regions except Somali.[9] In India, except for the southern region, as well as in Pakistan, Nigeria, and South Sudan, ANC utilization rates are low.[17,34,48,49] Low ANC utilization in these areas may be due to historical, socioeconomic, and cultural conditions across these physical settings.

Urban residence of women was significantly associated with adherence to the recommended number of ANC visits. This finding is consistent with meta-analysis in Ethiopia.[59] In Nepal, urban women were 7 and 2 times more likely to make the recommended and fewer than

recommended number of ANC visits, respectively.[16] Researchers suggest the reasons for the higher use might be the better quality of care and greater accessibility to professionals in urban areas. Non-significant association between urban residence and fewer than recommended number of ANC visits has been reported elsewhere.[31,48]

Our study had a number of limitations. All data were self-reported by the women participants and were not triangulated with other sources, which may have resulted in bias. The study also did not capture institution-based factors such as health providers' behavior and accessibility and quality of services, all of which influence the health-seeking behavior of women. Moreover, the long recall period may have introduced information bias.

Conclusion

Despite the fact that ANC attendance impacts safe motherhood and reduces maternal deaths, this study found unacceptably low adherence to the recommended number of ANC visits in rural western Ethiopia. Only 15.2% of women studied made the recommended number of visits to ANC clinics, 49.6% made fewer than the recommended number, and 35.2% did not obtain any ANC. The results of this study confirm the importance of several factors in women's making the WHO-recommended number of visits to ANC clinics: number of births, occupation, visits by HEWs, household income, headache, fever, decentralized administrative *kebele*, and urban-rural location. Religion, gendered decisions about resource use, walking time to ANC services, decentralized facility type, and vaginal bleeding were significant predictors of fewer than the recommended number of ANC visits.

These results indicate that the study of broadly-based interventions considering the socioeconomic, cultural, demographic, and environmental context of communities may be useful in identifying barriers to ANC utilization and promoting adherence to the recommended number

of maternal visits in rural western Ethiopia. Health campaigns conducted through *kebele* and *woreda* health services as well as mass media may promote health-seeking behavior of pregnant women and increase the awareness of communities, religious leaders, and other stakeholders about the recommended number of ANC visits. However, policies and programs must extend beyond community awareness of the need for adequate and appropriate maternal care and address also long-term multi-sectoral development issues. Broadly based interventions need to focus on motorized rural roads, public transport, livelihoods and income generation, and gender equity. Interventions must increase the number and the coverage of home visits by HEWs and upgrade and equip front-line health posts or further extend the decentralization of health centers in rural areas. These actions will ensure that the predisposing, enabling, need, and external environmental factors that promote health-seeking behavior are in place to achieve increased ANC utilization and reduction in maternal deaths.

Funding

The research was financially supported by Department of Geography and Environmental Studies of Addis Ababa University and Wollega University. The funders had no grant numbers. The funders had no responsibility in the study design, data collection, analysis and interpretation, and preparation of the manuscript for publication.

Acknowledgements

We thank Wollega University for providing us the ethical clearance to undertake the study. We also thank Gida Ayana *Woreda* health officials and the *kebele* administrators for their cooperation during data collection. Our special thanks go to the women participants for their

dedication and commitment in providing information. We also want to thank Mrs. Ann Byers for editing the manuscript.

Data Availability

The data are all contained within the manuscript and/or Supporting Information files

Declaration of conflicting interests

The authors declared no potential conflicts of interest with respect to the research, authorship, and/or publication of this article.

References

1. World Health Organization. *Trends in Maternal Mortality, 1990 to 2013. Estimates by WHO, UNICEF, UNFPA, World Bank and United Nations Population Division. Geneva, WHO*; 2014.
2. Woldegiorgis AM, BhowmikJ, Mekonnen W. Trends in reproductive health indicators in Ethiopia:2000-2014. *Int J Health*. 2017;3(1). doi:10.5430/ijh.v3n1p17.
3. Tessema AG, Laurence OC, Melaku AY, Misganaw A, Woldie AS, Hiruye A et al. Trends and causes of maternal mortality in Ethiopia during 1990-2013: Findings from the Global Burden of Diseases Study 2013. *BMC Public Health*. 2017;17(160). doi:10.1186/s12889-017-4071-8.
4. World Health Organization. *World Health Statistics 2015*. Geneva, WHO; 2015.
5. Central Statistical Agency and ORC Macro. *Ethiopia Demographic and Health Survey 2011.* Addis Ababa, CSA and Calverton, Maryland, USA, ORC Macro; 2012.
6. Central Statistical Agency and ORC Macro. *Ethiopia Demographic and Health Survey 2005.* Addis Ababa, CSA and Calverton, Maryland, USA, ORC Macro; 2006.

7. Ethiopian Federal Ministry of Health. *Health Sector Development Program IV. 2010/2011-2014/2015.* Addis Ababa, FMoH; 2010.

8. Ethiopian Federal Ministry of Health. *Health Sector Transformation Plan (2015/16-2019/20)*. Vol 20. Addis Ababa, FMoH; 2015.

9. Central Statistical Agency and ORC Macro. *Ethiopia Demographic and Health Survey 2016.* Addis Ababa, CSA and Rockville, Maryland, USAORC Macro; 2017.

10. Alkema L, Chou D, Hogan D, Zhang S, Moller A, Gemmill A et al. Global, regional, and national levels and trends in maternal mortality between 1990 and 2015, with scenario-based projections to 2030: A systematic analysis by the UN Maternal Mortality Estimation Inter-agency Group. *Lancet*. 2016;387(10017):462-474.

11. Harvey SA, Ayabaca P, Bucagu M, Djibrina S, Edson WN, Gbangbade S *et al*. Skilled birth attendant competence: An initial assessment in four countries, and implications for the safe motherhood movement. *Int J Gynecol Obstet*. 2004;87:203-210.

12. Thaddeus S; Maine D. Too far to walk : Maternal mortaliity in context. *Soc Sci Med*. 1994;38(8):1091-1110.

13. World Health Organization. Maternal Mortality: Factsheet. 2018. Retrieved from: http://www.who.int/ mediacentre/factsheets/fs348/en/. 2018:2018.

14. Koblinsky M, Tain F, Gaym A, Karim A, Carnell M, Tesfaye S. Responding to the maternal health care challenge: The Ethiopian health extension program. *Ethiop J Health Dev*. 2010;24(Special Issue 1):105-109.

15. Pell C, Menaca A, Were F, Afrah NA, Chatio S, Taylor ML et al. Factors affecting antenatal care attendance: Results from qualitative studies in Ghana , Kenya and Malawi. *PLoS One*. 2013;8(1). doi:10.1371/journal.pone.0053747.

16. Shrestha G, Shrestha G. Statistical analysis of factors affecting utilization of antenatal care in Nepal. *Nepal J. Sci Technol*. 2011;12:268-275.

17. Singh PK, Rai RK, Alagarajan M, Singh L. Determinants of maternity care services utilization among married adolescents in rural India. *PLoS One*. 2012;7(2). doi:10.1371/journal.pone.0031666.

18. United States Agency for International Development. *Cultural Barriers to Seeking Maternal Health Care in Ethiopia. A Review of the Literature: Ethiopia.* Washington, DC. USAID; 2012.

19. World Bank. *World Bank Country Status Report on Health and Poverty in Ethiopia.* Washington, DC., Africa Region Human Development and Addis Ababa, Ministry of Health Ethiopia; 2004.

20. Tolera H, Gebre-Egziabher T, Kloos H. Public health service delivery in a decentralized system: A qualitative study of the perception of health providers and community members in Gida Ayana Woreda, western Ethiopia. *Glob Journals Med Res*. 2019;19(2):22-37.

21. Kassa A, Shawel Y. Integrating all stakeholders: Health service governance in Addis Ababa. In: Chanies P, Mihyo P, eds. *Thirty Years of Public Sector Reforms in Africa:Selected Country Experiences*. Kampala, Fountain; 2013:55-131.

22. Federal Ministry of Health. HSDP III: Ethiopia Health Sector Development Programme Mid-Term Review. Addis Ababa; 2008.

23. Aregay A, Alemayehu M, Assefa H, Terefe W. Factors associated with maternal health care services in Enderta district, Tigray, northern Ethiopia: A cross sectional study. *Am J Nurs Sci*. 2014;3(6):117-125.

24. Dulla D, Daka D, Wakgari N. Antenatal care utilization and its associated factors among

pregnant women in Boricha district, southern Ethiopia. *Divers Equal Health Care*. 2017;14(2):76-84.

25. Bitew T, Charlotte H, Kebede E, Medhin G, Fekadu A. Antenatal depressive symptoms and maternal health care utilisation: A population-based study of pregnant women in Ethiopia. *BMC Pregn Childbirth*. 2016;16(301):1-11. doi:10.1186/s12884-016-1099-1
26. Tesfaye G, Loxton D, Chojenta C, Semahegn A, Smith R. Delayed initiation of antenatal care and associated factors in Ethiopia: A systematic review and meta-analysis. *Reprod Health*. 2017;14(150):1-17. doi:10.1186/s12978-017-0412-4
27. Tiruaynet K, Muchie KF. Determinants of utilization of antenatal care services in Benishangul Gumuz Region, western Ethiopia: A study based on demographic and health survey. BMC Pregn Childbirth, 2019; 19(115). doi.org/10.1186/s12884-019-2259-x.
28. Tewodros B, Gebre-Mariam A, Dibaba Y. Affecting antenatal care utilization in Yem Special *Woreda* , Southwestern Ethiopia. *Ethiop J Health Sci*. 2009;19(1):45-50.
29. Phillips KA, Morrison KR, Andersen R, Aday LA. Understanding the context of healthcare utilization: Assessing environmental and provider related variables in the behavioral model of utilization. *Health Serv Res*. 1998;33(3):571-596.
30. Andersen RM. Revisiting the behavioral model and access to medical care: Does it matter? *J Health Soc Behav*. 1995;36:1-10.
31. Ayele DZ, Belayihun B, Teji K, Ayana DA. Factors affecting utilization of maternal health care services in Kombolcha District, Eastern Hararghe Zone, Oromia Regional State, Eastern Ethiopia. *Int Sch Res Not*. 2014; doi.org/10.115512014/917058.
32. Kumar A, Singh A. Explaining the gap in the use of maternal healthcare services between social groups in India. *J Public Health*. 2015:1-11. doi:10.1093/pubmed/fdv142.

33. Andersen RA. *Behavioral Model of Families' Use of Health Services.* Chicago, Center for Health Administration Studies, Research Series;1968.

34. Mugo NS, Dibley MJ, Agho KE. Prevalence and risk factors for non-use of antenatal care visits: Analysis of the 2010 South Sudan household survey. *Pregn Childbirth* 2015;15(68):1-13. doi:10.1186/s12884-015-0491-6.

35. Tarekegn MS, Lieberman SL, Giedraitis V. Determinants of maternal health service utilization in Ethiopia: Analysis of the 2011 Ethiopian Demographic and Health Survey. BMC *Pregn Childbirth*. 2014;14(161):1-13.

36. Central Statistical Agency. *Federal Demographic Republic of Ethiopia Central Statistical Agency Population Projection of Ethiopia for All Regions at Woreda Level from 2014 - 2017*. Addis Ababa, CSA; 2013.

37. Eastern Wollega Zone, Wone Finance and Economic Development Office. *Physical and Socio Economic Profile of Gidda Ayana Woreda*. Nekemte, Ethiopia; 2015.

38. Kelsey J, Whittemore A, Evans A, Thompson W. *Methods ofSampling and Estimation of Sample Size. Methods in ObservationalEepidemiology.* New York, Oxford University Press; 1996.

39. Central Statistical Agency. *Ethiopia Mini Demographic and Health Survey 2014.* Addis Ababa: Central Statistical Agency and Ministry of Healt. Ethiopia, 2014.

40. World Health Organization, *Strategies Toward Ending Preventable Maternal Mortality*. Geneva, WHO; 2015.

41. Pallant J. *Survival Manual: A Step by Step Guide to Data Analysis using SPSS for Windows*. 3rd ed. New York, McGraw Hill Open University Press; 2007.

42. Hosmer D LS. *Applied Logistic Regression*. 2nd ed. New York, John Wiley and Sons, Inc.;

2000.

43. Fenta M . Assessment of factors affecting utilization of maternal health care services in Ayssaita and Dubti towns, Afar Regional State, Northeast Ethiopia. MPH thesis, Addis Ababa University, Addis Ababa; 2005.

44. Umar AS. The use of maternalhealth services in Nigeria: Does ethnicity and religious beliefs matter ? *Public Health*. 2017;6(6). doi:10.15406/mojph.2017.06.00190

45. Chakraborty N, Islam M. A, Chowdhury R.I, Bari W, Akhter HH. Determinants of the use of maternal health services in rural Bangladesh. *Health Promot Int*. 2003;18(4):327-337.

46. Umar AS, Bawa SB. Antenatal care services utilization in Yobe State, Nigeria : examining predictors and barriers. *Int J MCH AIDS*. 2015;4(1):35-46.

47. Qi Z, Asli K, Yi G, Biao X. Knoweledge and attitude on materanal health care among rrural-urban migrant women in Shanghai, China. *BMC Womens Health*. 2009;9(5). doi:10.1186/1472-6874-9-5.

48. Umar S.A. *Use of Maternal Health Services and Pregnancy Outcomes in Nigeria*. Walden, Walden Dissertations and Doctoral Studies, Walden University Scholar Works; 2016.

49. Shahram MS, Hamajima N, Reyer JA. Factors affecting maternal healthcare utilization in Afghanistan: Secondary analysis of Afghanistan health survey 2012. *Nagoya J Med Sci*. 2015;77:595-607.

50. Azfredrick EC. Using Andersen's model of health service utilization to examine use of services by adolescent girls in south-eastern Nigeria. *Int J Adolesc Youth*. 2016;21(4):523-529.

51. Ahmed S, Creanga AA, Gillespie DG, Tsui AO. Economic status, education and

empowerment: Implications for maternal health service utilization in Developing Countries. *PLoS One*. 2010;5:16. doi:10.1371/journal.pone.0011190

52. Kloos H. Utilization of selected hospitals, health centers and health stations in central, southern and western Ethiopia. *Soc Sci Med*. 1990; 31:101-114.

53. Erulkar, A. S, Onoka, C. J., Phiri A. What is youth-friendly? Adolescents' preferences for reproductive health services in Kenya and Zimbabwe. Afr. *J Reprod Health*. 2005;1:2051-2505.

54. Nathan LM. Shi Q, Plewniak K, Zhang C, Nsabimana D, Sklar M et al. Decentralizing maternity services to increase skilled attendance at birth and antenatal care utilization in rural Rwanda: A prospective cohort study. *Matern Child Health J*. 2015;19(9):1949-1955.

55. Alvarez FN, Leys M, Me H, Guzma GE. Primary health care research in Bolivia : Systematic review and analysis. *Health Policy Plann*. 2016;31:114-128.

56. Qureshi NR, Sheikh S, Khowaja RA, Hoodbhoy Z, Zaidi S, Sawchuck D. Health care seeking behaviours in pregnancy in rural Sindh, Pakistan: A qualitative study. *Reprod Health*. 2016;13(34):75-97.

57. Abosse Z, Woldie M. Original article factors influencing antenatal care service utilization in Hadiya Zone. *Ethiop J Health Sci*. 2010;20(1).

58. Majumder A. Utilisation of health care in north Bengal: A study of health seeking patterns in an interdisciplinary framework. J Soc Sci 2006;13(1):43-51.

59. Mekonnen T, Dune T, Perz J, Ogbo FA. Trends and determinants of antenatal care service use in Ethiopia between 2000 and 2016. *Int J Environ Res Public Health,*. 2019;19(74). doi:10.3390/ijerph16050748

Fig. 1 Conceptual framework of the study

Fig_1

Click here to access/download;Figure;Fig_1.docx

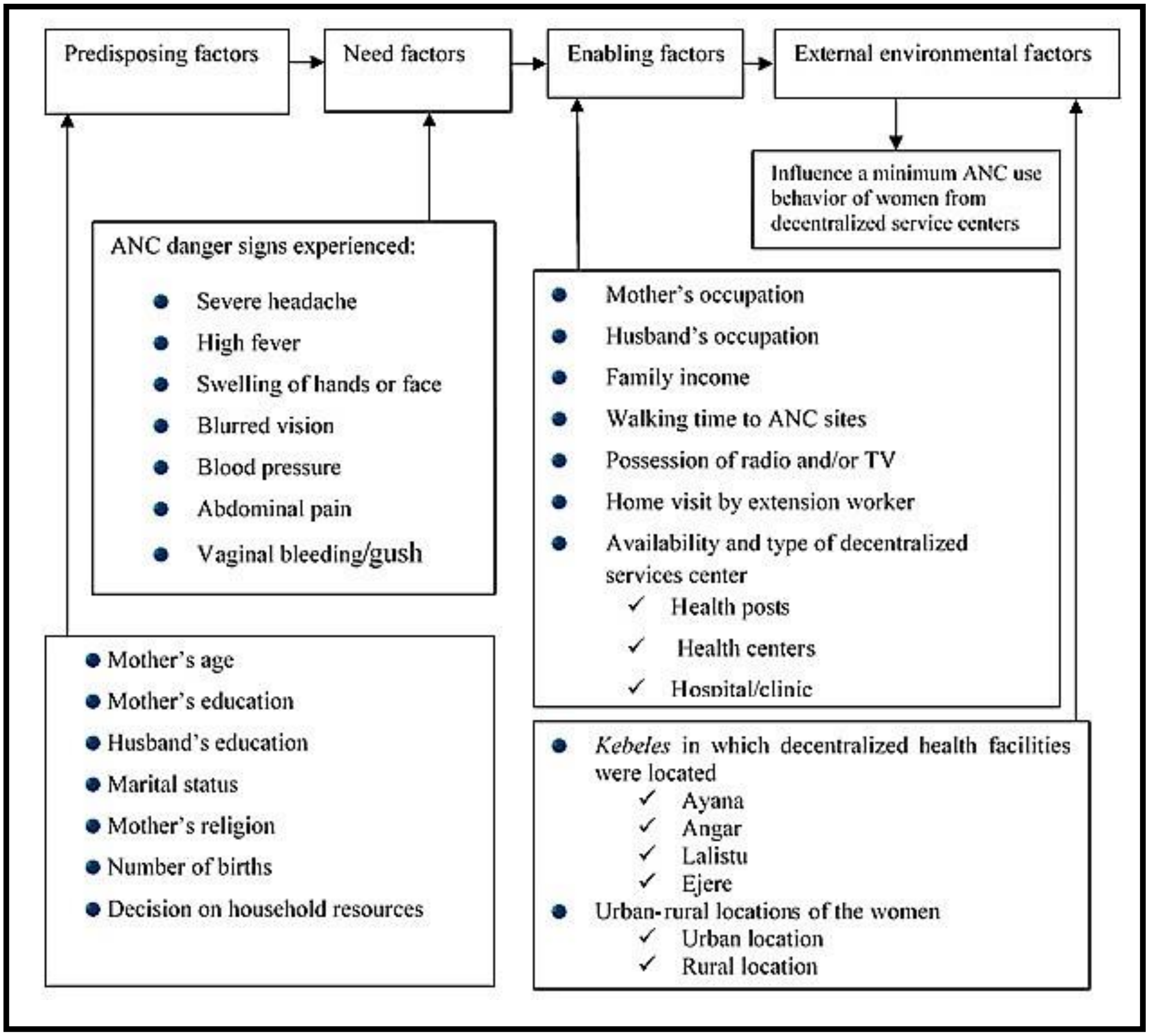

Anderson's original model

Click here to access/download;Figure;Fig_2 original Andersons Framework.docx

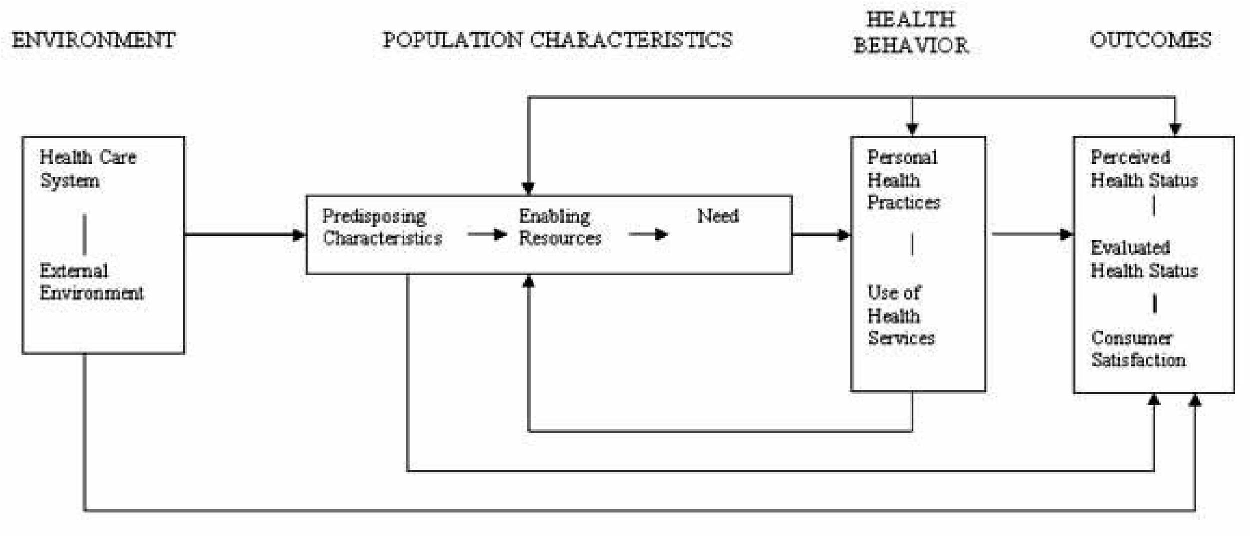

Supplement: Attachment — Submitted filename: PONE-D-19-25468-Metadel comments.pdf [file pone.0228282.s003.pdf]
