# Supplementary material for: Using Andersen’s behavioral model of health care utilization in a decentralized program to examine the use of antenatal care in rural western Ethiopia
Source: PLoS One. 2020 Jan 27;15(1):e0228282. doi: 10.1371/journal.pone.0228282 (PMC6984696; doi:10.1371/journal.pone.0228282)
Supplement: Attachment — Submitted filename: PONE-D-19-25468-comment.doc [file pone.0228282.s004.doc]

## Dear Prof Kannan Navaneetham

Thank you for inviting me to review this interesting paper. I have a very minor collection of comments and hoping my comments are very easy for the author to make correction and I recommend you to accept the paper without modification.

**Title**

Q1. Title: Using abbreviation in title ANC alone is confusing, please write both the expansion and the abbreviations?

Q2. The short title is not actually short: I recommend this, Health care utilization and antenatal care Services in rural western Ethiopia.

**Abstract**

Q3. Abstract: Re-write the methods and result section separately for clarity to readers, please see my comments within the PDF

Q4. From the abstract result section, for all AOR, please include the confidence interval to see how the association is strong or weak? This is very key information for the scientific community.

Q5. From conclusion of the abstract, utilization of recommended ANC services was strongly linked with predisposing, enabling, need, and external factors? This seems very general better to mention those factors which are actually associated with among predisposing, enabling, need, and external factors due to all factors of predisposing, enabling, need, and external factors are not actually a factor for the use of ANC?

Q6. From line 34, which intervention you recommended as a researcher?

Q7. Key word is not applicable for PLoS ONE publication, thus, delete it.

**Introduction**

Q8. Referencing style should be in line with the PLoS OEN guideline? This comment for all throughout your MS.

Q9. Line 46-50 should be replaced by the current HSTP (Health Sector Development Plan) and SDG goals, why you sued the expired MDG and HSDP information?

Q10. Better to delete lines 81-83 since it is repetition with line 93-94. Keeping line 93-94 idea is good.

**Methodology**

Q11. Better to put the map of the study area, if the author put the study area in another published of his paper, he can cite that paper without putting the map within this paper. This gives a clear view for the readers of this paper.

Q12. Line 100 and line 102 refrence should be corrected. Use CSA reference for line 102 AND LINE 100 for Eastern Wollega Zone, Wone Finance and Economic Development Office. *Physical and*

561 *Socio Economic Profile of Gidda Ayana Woreda*. Nekemte, Ethiopia; 2015. Making both at the same time seems both data available in both sources, which could not be in real situation?

Q13. Line 104, better to write a ‘community based’ than population based. Again, the langue is not clear, Please see my comment within the PDF for line 104.

Q14. Line 110 to 112 had confusing ideas, if the source population less than 10,000, sample size correction is done and adding 5% non-response rate does not the justification. Adding a non-response rate either less than or greater than 10,000 is a must. Please re-write it/

Q15. Line 121 of using recent births, why not you select randomly one of the mother?

Q16. Line 125-126 seems it will incur bias? Btter to delete it.

Q17. Line 131. Cite the WHO recommendation source for ANC categorization?

Q18. Line 136 to 162 should not be one paragraph? Make it at-least 3

Q19. Line 165-66, re-write, What does it mean literature? Did you mean published paper

Q20, the full name and abbreviation for health extension workers written wt line 117, then at line 170 use only the abbreviations, such comment works for all. Once you used both the full name and the abbreviation, then use the abbreviation, check also about EDHS, ANC and etc.

Q21. Line 181 and 812, re-write for clarity.

Q22 Line 178-193, make it at least two paragraph?

Q23. Line 194. consent is part of ethics and no need to write as a topics , make it the title ‘Ethical consideration”

**Result**

Q24. Line 219, The exchange rate should be during your data collection period? And please write the time from your result display or in the table?

Q 25. Line 220, Table 1 should have information about mean age, and mean number of children women gave birth? Putting by text from line 211 is not enough?

Q26. Table 1 topic needs modification. There is no cultural related data in the table so that saying Socio-cultural and demographic backgrounds not correct? But the Table also have HEWs information then, you can find out better title as reflecting what exist in the Table?

Q27. For all Tables write the study period and the correct location of the study area by including rural western Ethiopia.

Q28. Liens 236-238 should be deleted since the bivariate association has no value? But I understand that Table 3 about multivariable analysis result and if so, the Table 3 title saying bivariate is total wrong and confusing? Please check

Q29. If Table 3 about Bivaraite analysis, your note on 235 to 307 should be deleted since bivariate data is noting for decision making and the association have no value.

**Discussion**

Q30. Please bring Table 4 above the discussion at result section?

Q31. Discussion better to be re-written? Repeating the result section at the discussion is not recommended, either you use result and discussion within the same topic or separately, I thought you have a separate topic for discussion, so in your discussion section focus on discussions part? No need to write each result again? At the result section, put all your result and focus on the main findings, you wasted your time at the result by bivariate result at Table 4, but the most important finding is Table 4.

Q31. Line 451, write the reason why no grant number

Q 32, 455, in your acknowledgement, please acknowledge also data collectors supervisors, Addis Abbaa University, Department f Geography at AAU and etc. This tells how you are very careful
